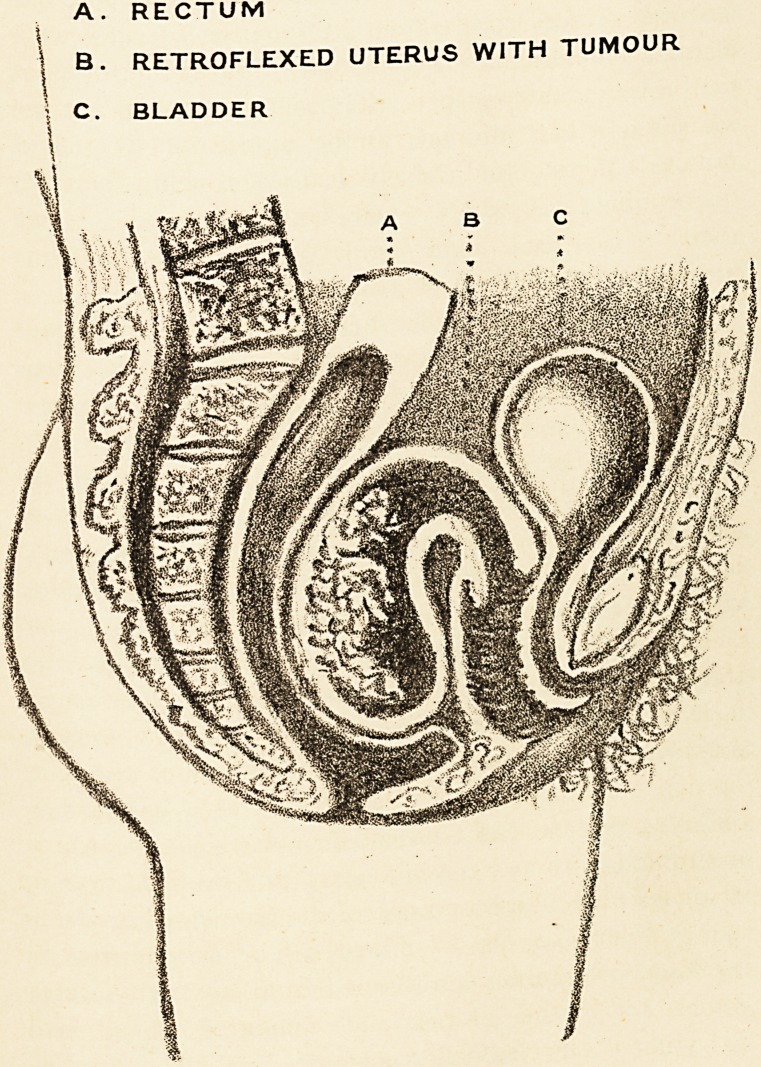# Case of Epithelioma of the Body of the Uterus

**Published:** 1890-06

**Authors:** J. G. Swayne

**Affiliations:** Consulting Physician-Accoucheur to the Bristol General Hospital


### CASE OF EPITHELIOMA OF THE BODY OF THE UTERUS. By J. G. Swayne, M.D., Consulting Physician-Accoucheur to the Bristol General Hospital.*

The chief points of interest in this case are—first, its insidious character; second, its long duration.

The patient (Mrs. B.), from whom this specimen was taken, first consulted me in 1882. She was then 59 years of age, and tolerably healthy in appearance. She consulted me for a dull, aching pain in the sacral region, increased by standing or walking. She had previously been under the care of another medical man, who considered the pain to be due to rheumatism and treated her accordingly. As the pain was not relieved by the remedies used, her friends persuaded her to consult me

* Specimen shown and case read before *Bristol Med. Chir. Soc.*

about it, with the object, principally, of her undergoing an internal examination. On examining her I found the uterus to be healthy in size and appearance, such as one would expect to find in a multipara past the menopause. It was, however, considerably retroflexed, but quite movable, and I had no difficulty in passing a sound and reducing it. I then introduced a Hodge's pessary, with a cushion of india-rubber filled with glycerine at the sacral end; and this gave her so much ease and comfort that she was able to walk about without pain, and, in fact, believed herself to be cured. Although suffering occasionally from the displacement, for the next few years she went on, on the whole, very well, as shown by my visits to her, which in 1883 were only three, none in 1884, eight in 1885, and thirteen in 1886. At the end of 1886, however, a change for the worse seemed to take place, for in 1887 my visits to her mounted up to forty-seven. The fact was that towards the end of 1886 a change had taken place in her general condition. She had become somewhat emaciated, and showed symptoms of constitutional irritation. The uterus instead of shrinking had become larger, especially in the body, and, pressed back more into the pouch of Douglas, it was very irritable—so much so, that I dared not use the sound to replace it; the os was more open, and admitted the tips of the fingers readily, and a thin slightly sanguineous discharge issued from it—which, however, was not offensive. Thinking that it was a case of catarrhus senilis of the uterus, I used periodically uterine injections of nitrate of silver, ten grains to the ounce. This had a very good effect for a time, and there was a good deal of improvement during the year 1888.

She, however, never entirely lost the symptoms from which she suffered, and in May, 1889, had become much worse, was greatly emaciated, and very despondent about her general condition—so much so, that her friends wished her to go to London for further advice. To this I readily assented, and she accordingly went up to London and consulted Dr. Mathews Duncan. He took much the same view of her case as I did—viz., that it was a case of senile catarrh,—and used uterine injections of nitrate of silver, but of about treble the strength of those which I employed. This benefitted her very much for a time—so much so, that she was able to walk in the Park, and thought herself really cured. Dr. Duncan, however, was not of that opinion; but encouraged by the good effect of the former injections, used one of double their strength. This gave her so much pain, and aggravated her former symptoms so much, that she would not go to him again, but consulted Dr. Priestley. He tried several intra-uterine injections, with some temporary good effect, but no permanent benefit; until at last, with his concurrence, she decided to return home on June 24th last, on which day I received a letter from him, in which he says: "I had great hope, by the way it seemed to yield to intra-uterine injections of tannin and glycerine and of tincture of iodine alternately, that we had only a case of senile catarrh to deal with; but the result of an injection on Friday last raises a suspicion in my mind that there is graver mischief in the uterine cavity. I introduced a silver syringe about two or two and a-half inches within the cervix, with great gentleness, and it gave her no pain, but a gush of hæmorrhage came on immediately, and so severely that I had to put a tampon in the vagina. Since then there is more discharge, again muco-purulent and bloody, and I fear there is an ulcerated surface producing this. Under the circumstances, I think I should scarcely venture to pass a tube into the uterine cavity again at present, but be content with passing a small cotton-wool tampon up to the os uteri, through a small speculum. The tampon can be dipped in glycerine of tannin or matico, and the glycerine—through its capacity of running everywhere—will permeate to the womb cavity. Besides this, a vaginal injection of chloralum in solution might be used every day."

I saw her the day after her return, and was shocked to see the rapid change for the worse in her appearance. Such extreme emaciation had set in, that she was reduced almost to a skeleton; she suffered from extreme mental depression; her stomach was so irritable that she could take very little food; and, in fact, she appeared to be gradually dying of asthenia. Her extreme emaciation brought out a fact which I had not noticed before, and to which she had never directed my attention; viz., that there was very great malformation of the chest, apparently the result of tight-lacing in youth. The cartilages of the false ribs were pressed close together, and the sternum much depressed; so that the heart was pushed out of its normal position to such a degree that its apex could be felt pulsating beneath the left shoulder-blade, instead of in front. Its impulse was very weak, but there was no distinct bruit. The condition of the uterus was much the same as when I last examined her before she went to London. I, therefore, abstained from any active interference with the uterus, and contented myself with carrying out the treatment recommended by Dr. Priestley, more especially in the use of the tannin and glycerine tampons.

A. RECTUM

B. RETROFLEXED UTERUS WITH TUMOUR

C. BLADDER

After seeing her, I pointed out to her relatives her extremely critical condition, and advised a consultation with some physician who might assist me in devising some means of improving the heart's action and nourishing the body more effectually. In accordance with their wishes, Dr. Shingleton Smith was called in, and for the first indication prescribed digitalis, and for the second, the use of peptonised foods. I had not many opportunities of consulting with him, for I had made arrangements to go away at that time for my annual holiday, which I accordingly did, my brother acting as my *locum tenens:* but before I had been absent three weeks I heard of her death; and on my return home I learned that Dr. Shingleton Smith and my brother had attended her to the last, and had finally made a *post mortem* examination, the result of which I now exhibit. The preparation has been preserved in spirit, and has consequently lost its colour; but it shows very well the shape and position of the uterus, and the extent of the intra-uterine tumour. I have copied this as closely as I could, and have represented it in the accompanying diagram.* There were no secondary deposits, either in the parts immediately around the uterus, or in any other part of the body.

In the discussion which ensued, two or three members present suggested that the tumour was adenomatous in character, and might have been removed successfully at an earlier stage by the curette. I did not incline to that opinion; but as no microscopical examination had yet been made, I could not speak positively as to its character. It was, therefore, decided that portions of the tumour should be examined microscopically, by Dr.

* The preparation is now in the Museum of the Bristol Medical School.

Shingleton Smith and Mr. Munro Smith, and the result reported at the next meeting. This was accordingly done, and a good microscopical section, made by Dr. S. Smith, was exhibited at the meeting. They both agreed that the tumour was undoubtedly carcinomatous in character.

REMARKS.—With regard to the progress and treatment of this case, I may state that during the first four years—viz., from 1882 to the end of 1886—no symptoms but those of retroflexion were present; there were, therefore, no indications at that period for dilating the os uteri and exploring the uterine cavity. But about the end of 1886 there was a rapid change for the worse in her general condition, and it was then probably that the carcinomatous tumour began to grow. The body of the uterus had increased in size, and become very tender; it was firmly fixed in the pouch of Douglas, and any attempt to introduce the sound, or replace it in any other way, caused the most acute pain. The uterine neck was bent at an acute angle upon the body, and could not be straightened. Under such circumstances the introduction of tents to dilate the canal of the cervix would probably have given rise to very serious symptoms. I, therefore, contented myself with the use of injections and other palliative treatment. The same kind of treatment was carried out by those who saw her subsequently in London; and I feel convinced that at that time, judging from the state of the uterus after death, it would have been impossible to have removed any considerable part of the tumour by the curette. The bend in the uterine neck, even after it had been removed from the body, was so great that the canal could not be straightened without tearing the tissues. Dr. Priestley found, when using an intra-uterine injection, that the bent tube introduced for that purpose when it came in contact with the morbid growth gave rise to such severe hæmorrhage that he could only restrain it by the tampon; and—very wisely, I think—did not attempt any further operation. If he had done so, and had made attempts to remove any portion of the growth, in the weak state the patient then was, she would in all probability have bled to death before it could have been completed.

As the neck of the uterus was normal in its structure, and as the morbid growth was strictly limited to the body, the only operation by which the carcinomatous tumour could have been completely removed would have been by excising the body of the uterus. But such an operation in a person of the patient's age and enfeebled condition would most certainly have been attended with a fatal result, and was, therefore, out of the question.

Mr. Lawson Tait, who is as bold as any man in performing difficult and dangerous operations when there is a fair prospect of a favourable result, speaks in the following terms of removing the uterus for cancer:—

"The proposal to deal with cancer of the uterus by complete removal of the organ meets, I need hardly add, with my strong disapproval. My reasons are, that its primary mortality must always be heavy; and that the few cases in which the disease does not recur are clearly errors of diagnosis. Further, operations for a disease which gives unjustifiable secondary results have no place in good surgery. . . . As I like my work to be stable, I have always opposed this cutting out of the uterus for cancer; and my first judgment has been confirmed by the results."*

* *Diseases of Women and Abdominal Surgery*, Vol. I., p. 117.

## Figures and Tables

**Figure f1:**